# Correlative light and electron microscopy reveals fork-shaped structures at actin entry sites of focal adhesions

**DOI:** 10.1242/bio.059417

**Published:** 2022-11-21

**Authors:** Karin Legerstee, Jason Sueters, Tsion E. Abraham, Johan A. Slotman, Gert-Jan Kremers, Jacob P. Hoogenboom, Adriaan B. Houtsmuller

**Affiliations:** ^1^ Erasmus Medical Centre Rotterdam, Department of Pathology, Optical Imaging Centre, 3000 CA, Rotterdam, The Netherlands; ^2^ Delft University of Technology, Department of Imaging Physics, 2628 CD, Delft, The Netherlands

**Keywords:** Cell migration, Correlative microscopy, Electron microscopy, Fluorescence microscopy, Focal adhesions

## Abstract

Focal adhesions (FAs) are the main cellular structures to link the intracellular cytoskeleton to the extracellular matrix. FAs mediate cell adhesion, are important for cell migration and are involved in many (patho)-physiological processes. Here we examined FAs and their associated actin fibres using correlative fluorescence and scanning electron microscopy (SEM). We used fluorescence images of cells expressing paxillin-GFP to define the boundaries of FA complexes in SEM images, without using SEM contrast enhancing stains. We observed that SEM contrast was increased around the actin fibre entry site in 98% of FAs, indicating increases in protein density and possibly also phosphorylation levels in this area. In nearly three quarters of the FAs, these nanostructures had a fork shape, with the actin forming the stem and the high-contrast FA areas the fork. In conclusion, the combination of fluorescent and electron microscopy allowed accurate localisation of a highly abundant, novel fork structure at the FA-actin interface.

## Teaser

**Summary:** We reveal and quantitatively assess a novel fork structure in focal adhesions using correlative fluorescence and electron microscopy

## INTRODUCTION

Focal adhesions (FAs) are the main cellular structures to link the intracellular cytoskeleton to the extracellular matrix (ECM). They are typically several square micrometres in size (Yamada and Geiger, 1997; Geiger et al., 2001). On the membrane-facing side, the main FA components are integrins, transmembrane receptors that directly bind to the extracellular matrix (ECM). F-actin, also known as filamentous actin, forms the edge of the FA on the cytoplasm-facing side. In between integrins and actin, a large and diverse intracellular macromolecular protein assembly is present, for which over 200 different proteins have been reported (Zaidel-Bar et al., 2007a; Winograd-Katz et al., 2014). These include (trans)membrane receptors, other than integrins, adaptor proteins and many different signalling proteins such as kinases, phosphatases and G-protein regulators, which through post-translational modifications add significantly to FA complexity. FAs experience force, the strength of which depends on the combination of myosin-II contractility and the stiffness of the ECM. Because of their importance in cell adhesion and to the transmission of force from the cell to the extracellular matrix, FAs are crucial to most types of cell migration, including *in vitro* over a 2D-surface. Migration and adhesion are key cellular functions required for many physiological and pathophysiological processes, like embryological development, the functioning of the immune system and cancer, in particular metastasis (Wahl et al., 1996; Winograd-Katz et al., 2014; Maartens and Brown, 2015).

The F-actin associated with FAs takes the shape of stress fibres, a specialised form of actin associated with contractile myosin II and cytoskeletal proteins such as α-actinin (Burridge and Guilluy, 2016). There are two types of stress fibres associated with FAs: ventral stress fibres are associated with FAs at either end and typically transverse the whole cell, while dorsal stress fibres are linked to FAs on one end, typically near the cell front, then stretch upwards to the nucleus and the dorsal cell surface (Small et al., 1998).

Here, we examine FAs and their associated F-actin fibres using a correlative fluorescence microscopy and Scanning Electron Microscopy (SEM) approach. Because FAs are very dense protein complexes found at the very edge of the cell and directly attached to the ECM, they are well suited to studying with SEM. We used cells stably expressing a fluorescently tagged form of the major FA protein paxillin to mark the FAs and fluorescently tagged phalloidin to stain the F-actin network. Although FAs have frequently been visualised using SEM (Biggs et al., 2008b,a, 2011; Friedmann et al., 2011; Martinez et al., 2008; Richards and Gwynn, 1995; Richards et al., 2001), overlaying the SEM images with the fluorescence images creates the possibility to clearly mark the FA boundaries in the SEM images. This revealed that FAs have a higher contrast in these images at the tip where actin fibres enter. Further examination revealed that these high contrasting FA areas and the associated F-actin fibre together have a forked shape, with the actin forming the stem and the high contrast areas within the FA forming the fork. Since no contrast-enhancing staining agent was applied this shows that protein density and possibly also phosphorylation levels are increased at the fork.

## RESULTS AND DISCUSSION

Here we examine FAs and their associated F-actin fibres using correlative fluorescence and scanning electron microscopy. Indium-Tin-Oxide (ITO) coated glass coverslips were additionally coated with a thin layer of type-I collagen to mimic the ECM, providing a surface for the integrin receptors to bind to. The ITO-coated glass provides a conductive substrate, which allows SEM inspection of thin samples such as cultured cells without metal shadowing or other conductive coating and/or without additional staining (Pluk et al., 2009; Liv et al., 2013; Peddie et al., 2014; Voorneveld et al., 2014; Joosten et al., 2018; Pinotsi et al., 2019). Onto these coverslips human bone cancer (U2OS) cells were seeded, stably expressing GFP-tagged paxillin to visualise the FAs. The cells were given 36 h to adhere and form clear FAs, followed by chemical fixation (4% paraformaldehyde), permeabilization and staining with phalloidin to fluorescently stain the F-actin fibres. Immediately before imaging, the samples were dehydrated (ethanol). During imaging we were able to switch between fluorescent and SEM imaging without moving the stage (Liv et al., 2013). However, in vacuum, under these dehydrated, permeabilised conditions, the GFP signal was relatively weak (Peddie et al., 2014; Peddie et al., 2017). Therefore, we first made the fluorescent images then followed up with the SEM images. To create overlay images, the two images were scaled to the same size followed by a manual overlay procedure, mainly on the basis of the F-actin network, since owing to the phalloidin staining this is clearly visible in both imaging modalities. Of the 122 FAs with associated actin fibres, 97 were clearly visible in both modalities and the remaining 25 were unclear in the SEM images. These latter FAs were located further from the edge of the adherent part of the plasma membrane, where the cell is thicker. In these areas, the FAs were often lost in the signal from the rest of the cell including other fibre networks, as no specific markers or contrast agents were used and the SEM at the low energies used only penetrates the upper tens of nm of the sample.

**Fig. 1.**
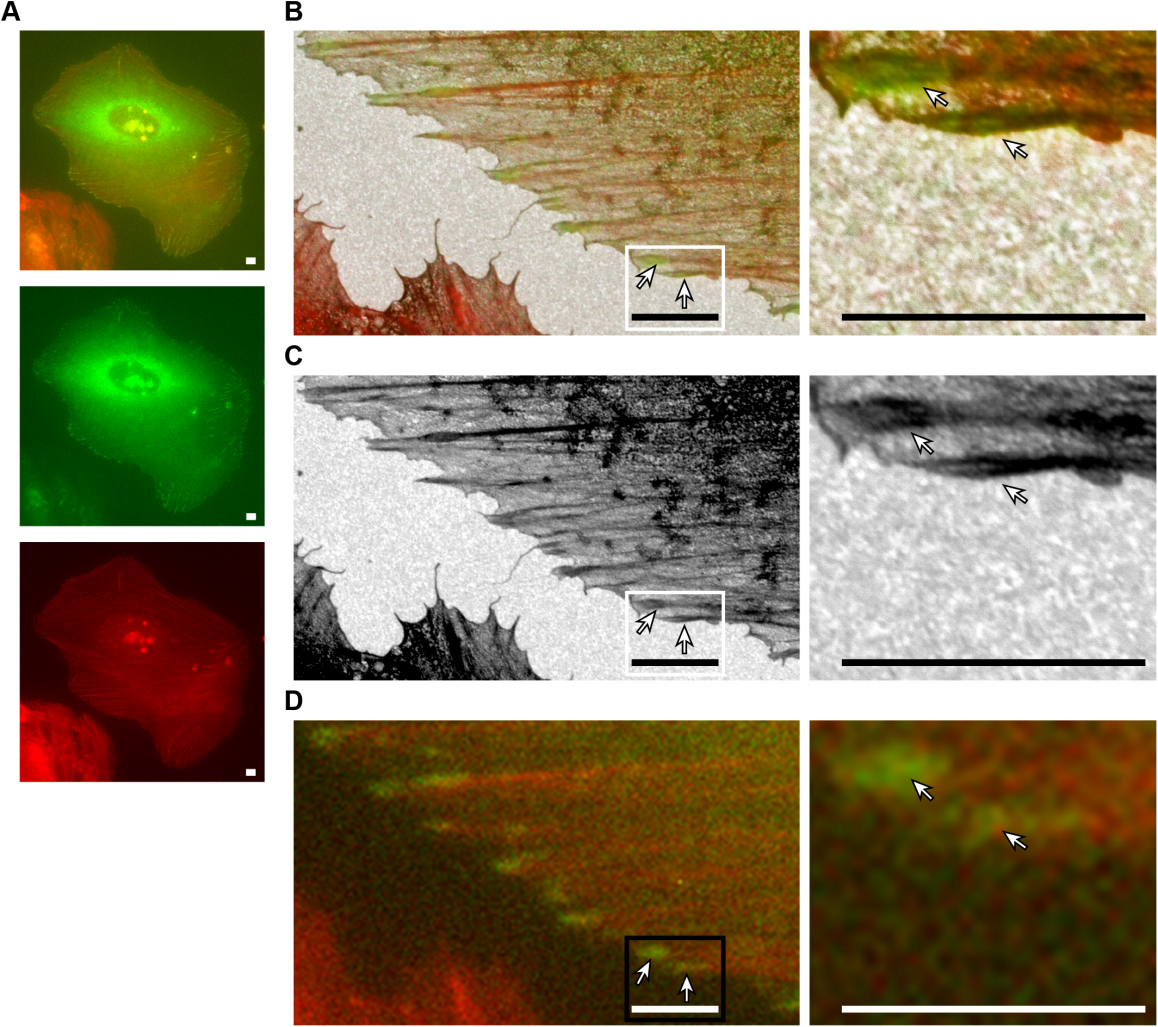
**Overlay of fluorescent and SEM images reveals high-contrast FA areas around the F-actin entry site.** Images of a U2OS cell stably expressing GFP-tagged paxillin and stained with phalloidin cultured on a collagen-coated ITO coverslip. (A) From top to bottom: merged image, green channel showing FAs where paxillin is localised and red channel showing phalloidin-stained F-actin. Scale bars: 5 µm. (B) Overlay image of a section of the fluorescent image in A with the corresponding SEM image (left) and zoom in of boxed area (right). Arrows indicate FAs with characteristic fork shapes. The fork is formed by higher-contrast areas of the FA that are partially intersected by areas of lower contrast, splitting the high-contrast areas into two sides. The stem is formed by the F-actin fibre entering the FA. Scale bars: 5 µm. (C) SEM channel of the image in B. (D) Fluorescent channel of the image in B.

**Fig. 2.**
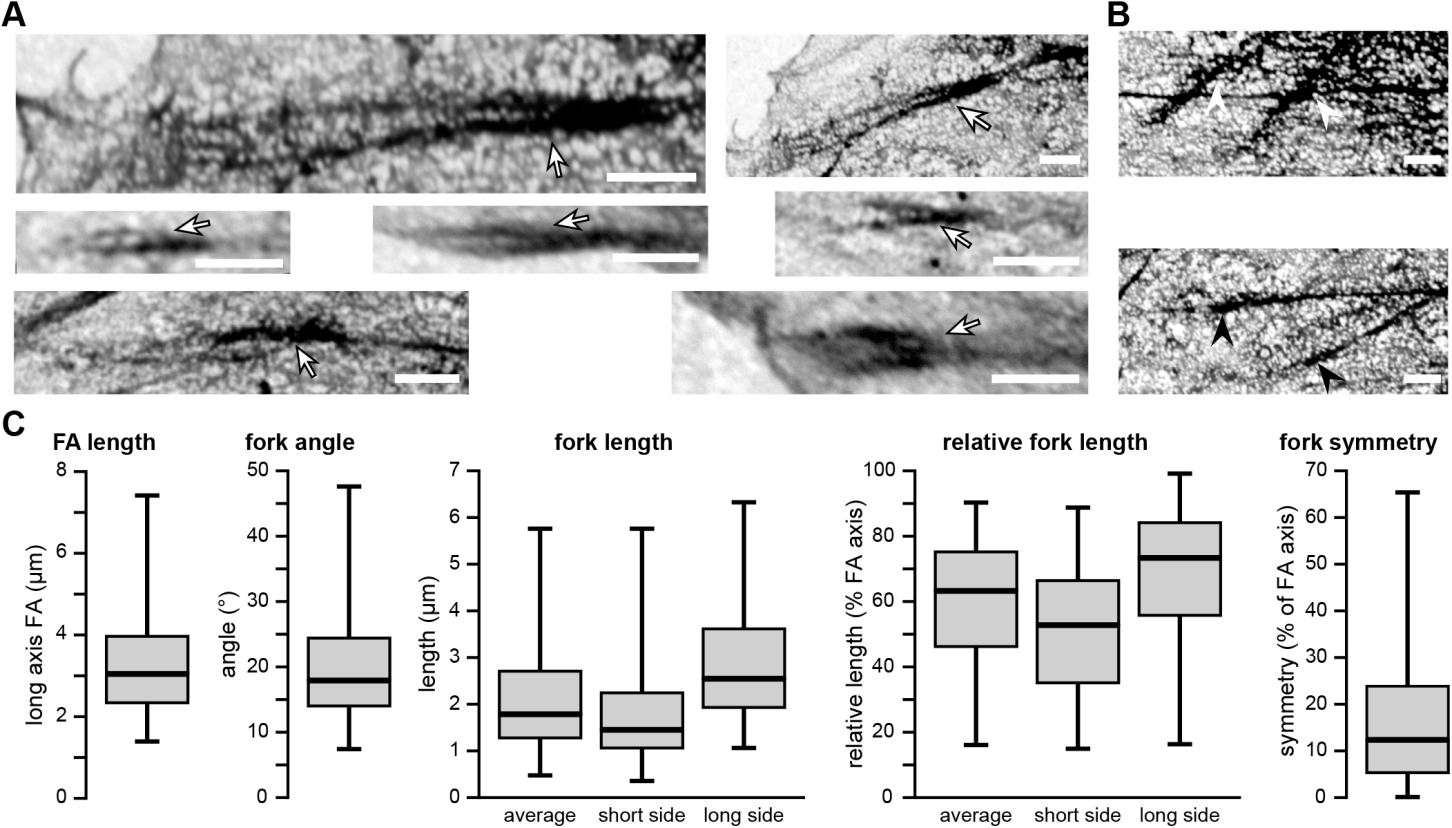
**Representative examples of fork-shaped, high contrast areas around the F-actin entry site**. (A) High-magnification SEM images of FAs in which the high-contrast areas around the F-actin entry site forms the characteristic fork-like shape (arrows). Scale bars: 1 µm. (B) Representative examples of high-magnification SEM images. Top panel: FAs excluded from the analysis because their signal is lost in the signal coming from the cytoplasm/cytoskeletal networks due to their more inward cellular position, making it impossible to accurately determine whether the high-contrast FA area is fork shaped or not (white arrow heads). Bottom panel: FAs classified as having high contrast areas not forming a fork shape (black arrowheads). Scale bars: 1 µm. (C) Results of a quantitative analysis of the observed fork shapes (73 FAs with fork shape from 10 cells) shown as box and whisker plots (horizontal line: median value, box: interquartile range, whiskers: median and maximum values). FA length shows the length of the long axis of the FA. Fork angle shows the angle between the two sides of the fork. For fork length, the length of the longest and shortest side of each fork was measured separately, as well as the average length of the two sides of each fork. For relative fork length the average, longest and shortest side of each fork was calculated relative to the corresponding FA length, with the long axis of the FA set to 100%. For fork symmetry, the average difference in length between the long and short side of the fork relative to the corresponding FA length was calculated.

**Fig. 3.**
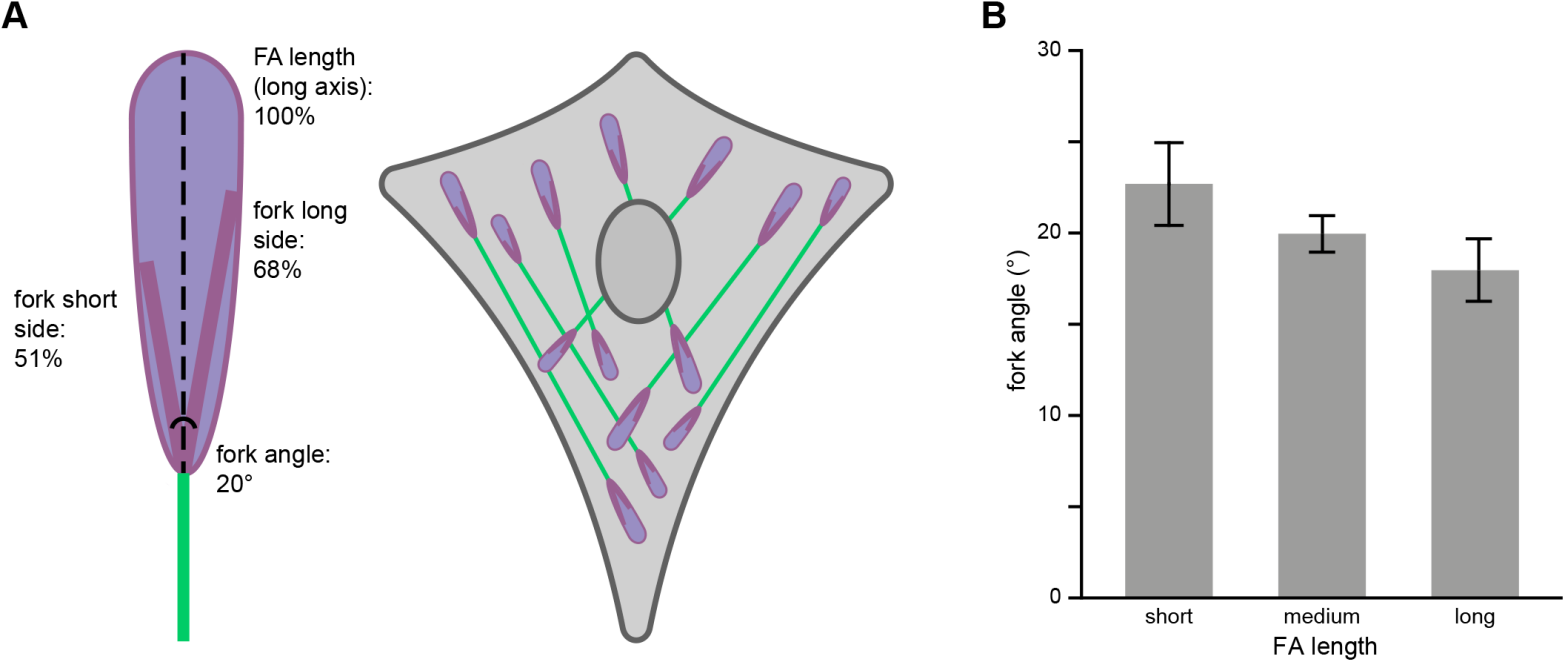
**The fork-shaped, high-contrast area of FAs around the F-actin entry site.** (A) Schematic representation of the average fork shape observed within FAs based on quantitative analysis of all observed fork shapes (see Materials and Methods) showing the average length of both sides of the fork and the average fork angle. (B) There is a trend for the angle between the two sides of the fork to decrease as the length of the FA increases (see Materials and Methods for details how these are defined and measured). For this analysis, the FAs were split into three groups: short, the 24 shortest FAs (ranging from 1.4-2.5 µm); medium, 24 FAs of medium length (ranging from 2.5-3.7 µm); and long, the 25 longest FAs (ranging from 3.7-7.4 µm). Error bars represent s.e.m.

FAs are visible in the SEM images but the overlay images are needed to clearly define the boundaries of the FAs (Fig. 1). Especially where the FA connects to its F-actin fibre it is almost impossible to determine from the SEM-images alone where the F-actin fibre ends and the FA begins. However, using the GFP-paxillin signal to mark the FA boundaries, it becomes clear that in the SEM images the contrast of the FA complex is increased around the entrance point of the F-actin fibre. The SEM images show an apparent reverse contrast due to the comparatively high backscatter electron yield of the indium- and tin-containing sample substrate, so areas with less transmission and thus more electron-scattering biological material appear as darker areas, as first noted by Pluk et al. (2009). Moreover, the overlays show that the high contrast areas are not part of the actin fibre but of the FA, as identified on the basis of the paxillin fluorescent signal. Such high-contrast areas were observed in 98% (95 FAs) of FAs (Table 1). Further examination showed that in almost three quarters of FAs, the high-contrasting areas and the associated F-actin fibre are fork shaped, with the actin as the stem and the high-contrasting FA areas as the fork (Fig. 2). High-contrasting areas within the focal adhesion (identified by the presence of paxillin fluorescence) were defined as forked when they were partially intersected by an area of lower contrast splitting the high-contrast area into two sides (fork). The angle between the two sides of the fork varied between 7 and 48 degrees, with an average angle of ∼20±2.0 degrees (±twice SEM). The average length of the fork was roughly 2 micrometres, which corresponded to ∼60±4.5% of the long axis of the FA (Fig. 2C). Looking at the two sides of the fork individually, the shortest side was on average ∼51±4.8% of the longitudinal FA axis (1.7±0.03 µm) and the longest side ∼68±4.8% (2.3±0.03 µm) (Figs 2C and 3A). With regard to symmetry, the average difference between the lengths of the two sides of the fork was 17.0±3.5% of the FA axis, with a minimum difference of only 0.1% and a maximum difference of 90.3%. Lastly, there was as a trend for longer FAs to have a smaller angle, suggesting the angle of the forks might decrease as FAs mature and grow (Fig. 3B).

**Table 1.**
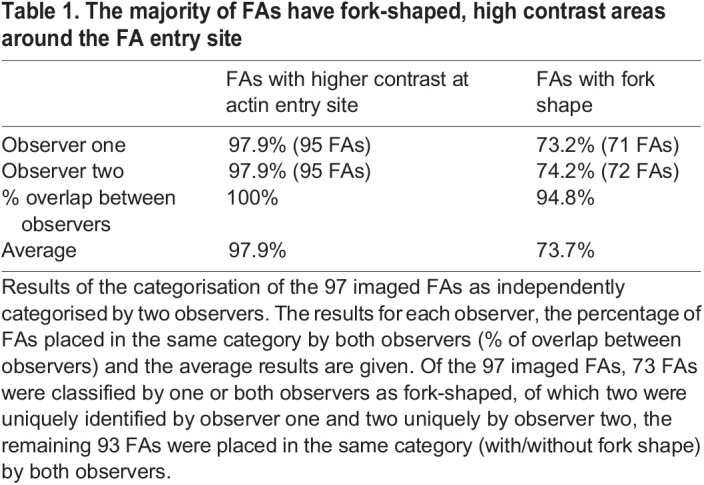
The majority of FAs have fork-shaped, high contrast areas around the FA entry site

To our knowledge, this is the first report on forked structures with high contrast seen in FAs, possibly because when using correlative fluorescent images the FA boundaries are clearly defined, whereas based on the SEM images alone the high contrast areas might easily be mistaken for parts of the actin fibre. However, with this new knowledge of exactly what to look for, we were now able to identify (hints of) the fork shape in previously published EM images of FAs, that were apparently not recognized at that time (Medalia and Geiger, 2010; Biggs et al., 2008a; Shu et al., 2011). We also note that while we followed a sample preparation procedure aimed specifically at preservation of fluorescence, the other works used EM-specific sample preparation protocols, indicating that the fork shapes observed here are not an artefact of our fixation procedure. Furthermore, we also visualised the fork-shaped structure using expansion microscopy in cells grown directly on collagen-coated glass coverslips, indicating that the forks are not an artefact of the ITO-coating either (Fig. S1).

The nanoarchitecture along the z-axis of FAs has been well described previously (Kanchanawong et al., 2010). Three different layers have been recognised: (1) the so-called integrin signalling layer (ISL) closest to the adherent membrane (within ∼10-20 nm), which includes the cytoplasmic tails of the transmembrane integrin receptors, focal adhesion kinase and paxillin, (2) the actin-regulatory layer (ARL) at the top, where mainly directly actin-binding proteins such as zyxin, vasodilator-stimulated phosphoprotein (VASP) and α-actinin are found, and (3) the force transduction layer (FTL) in between (from ∼10-20 to ∼50-60 nm from the adherent membrane) of which talin is the most well known.

Along the longitudinal axis of FAs, different proteins (vinculin, zyxin, paxillin and integrins) were shown to form nanoclusters, suggesting the FA complex as a whole might also be composed of discrete nanoclusters (Betzig et al., 2006; Shroff et al., 2007; Deschout et al., 2017; Xu et al., 2018; Spiess et al., 2018). Our data confirms heterogeneity along the longitudinal axis is a feature of the FA complex as a whole, since we are observing the FA complex in an unstained state instead of labelling a specific protein type. More importantly, we provide a location within the FA complex and a specific shape to this heterogeneity, namely a fork at the tip where the actin fibre enters the FA.

Other studies reported actin structures similar to the FA fork structure we report here. Note, however, that the fork-shaped structures we observed are in FA regions where no actin was present in the fluorescent images, indicating that the structures contain FA proteins and no or very little actin. In one study, the authors mapped the organisation of actin along the z-axis, which revealed that at FAs, vertical distribution of actin peaks at 80-120 nm from the extracellular matrix, which corresponds to a z-position just above the layered FA complex but there is overlap with the top ARL-layer (Liu et al., 2015). At higher z-positions, above the FA core, the actin was compacted and bundled into parallel stress fibres also enriched for α-actinin. In two other studies, the lateral axis FAs were shown to consist of repeating linear subunits about 300 nm in width (Hu et al., 2015; Young and Higgs, 2018). Different proteins from all three FA layers were examined, where all were organised in colocalising linear subunits with a width of 300±100 nm, while these functional FA proteins were absent from the space between the subunits. Also here, the organisation of actin was examined, and each linear subunit was found to be linked to individual actin radial cables of the same width. Interestingly, these linear subunits were associated with their individual actin fibres at their proximal tips but the actin did not always extend all the way to their distal tips. This may indicate that these actin cables contribute to the fork shape we observed by specifically increasing the protein density around the actin entry site. However, the average number of linear subunits and linked individual actin cables is six, more than the two sides of the fork we report here.

On a non-structural level, a few specific parameters have been shown to vary along the longitudinal axis of FAs, for example the binding dynamics of the hallmark FA protein paxillin as was shown in different studies using different approaches (Webb et al., 2003; Digman et al., 2008; Wolfenson et al., 2009; Legerstee et al., 2019). Traction forces and molecular tension of FA proteins are also non-uniformly distributed along the longitudinal axis (Plotnikov et al., 2012; Sarangi et al., 2017; Kumar et al., 2018). Surprisingly, maximum forces are not found around entry sites of actin fibres, but instead at the opposite tips of the FA. It has been shown that the level of paxillin phosphorylation decreases when force is increased and indeed the level of paxillin phosphorylation has also been demonstrated to vary along FAs (Zaidel-Bar et al., 2005; Nayal et al., 2006; Zaidel-Bar et al., 2007b). This could also be contributing to the formation of the observed high contrast areas, since here the force experienced by the proteins is the lowest, increasing the phosphorylation level of paxillin and perhaps other proteins as well. Strong local increases in the presence of slightly heavier elements than carbon, like phosphor, could further enhance the contrast in SEM images, in addition to a higher density of biological material. In this regard it is also interesting that in in certain FAs, mainly in very active FAs growing at the leading edge or sliding at the trailing edge of actively migrating cells, the intensity of talin staining is higher towards the cell centre (Kumar et al., 2018). This corresponds to the area where we find the fork shape in our mainly stably FAs in our non-migrating cells.

A final example of heterogeneity along the longitudinal axis of FAs involves the activity of vinculin. Vinculin is a large adaptor FA protein like paxillin, but it has a head and a tail domain connected by a flexible linker, allowing vinculin to adopt open (active) and closed (inactive) conformations (Bakolitsa et al., 2004). This allows assessment of vinculin’s activity levels through the use of a Foster resonance energy transfer (FRET) biosensor probe, which revealed that FAs at the retracting edge exhibit a gradient of vinculin activity along their lateral axis, with activity increasing towards the actin entry site (Chen et al., 2005). A later study was able to generalise these results to FAs beyond the retracting edge (Case et al., 2015). It showed that inactive vinculin associates with the FA layer closest to the adherent membrane (ISL) by binding to phosphorylated paxillin, while talin causes vinculin activation and a shift to the higher FA layers where it binds to actin. It was found that inactive vinculin in the ISL is significantly enriched at the FA tip where the actin fibre enters. As inactive vinculin binds to phosphorylated paxillin, this ties in well with studies showing that the forces at actin fibre entry sites are the lowest, leading to higher paxillin phosphorylation levels. The opposite FA tip is significantly enriched in activated vinculin located in the higher FTL and ARL layers, again demonstrating a vinculin activation gradient along FAs. However, our data show that heterogeneity along the longitudinal axis is a general feature of FAs and extends beyond the activity levels of a single protein like vinculin. Differing levels in one protein alone would not explain the increased contrast in our SEM images around the actin fibre entry site.

Based on the literature, next to paxillin and vinculin, α-actinin is another protein likely to be involved in the formation of the observed forked shape. In a publication using a genetically coded tagged version of α-actinin specifically designed for use with EM, a forked shape analogous to ours can be clearly seen but was not specifically mentioned (Shu et al., 2011).

Summarising, we show that the FA complex is altered around the actin fibre entry site leading to enhanced SEM contrast compared to the rest of the FA. In correlative fluorescence SEM images almost all FAs showed differential levels of contrast and in nearly three quarters of FAs this took the form of fork-shaped structures flanking the actin fibre entry point. Contrast is increased in SEM either when a structure is more dense or when more heavy elements are present, or both. Based on previous literature, it could be hypothesised that proteins likely to be involved in the formation of the fork-shaped structure are paxillin, which is more heavily phosphorylated around the actin entry site, vinculin, the inactive form of which binds to phosphorylated paxillin and is enriched around the entry site, and α-actinin, which increases density around the entry site by accumulating here into a fork-shaped structure. The fork-shaped structures reported here provide a clear avenue for future research, for example using the recently developed superresolution techniques (see Tapial Martínez et al., 2020; Mishra and Manavathi, 2021 for excellent overviews of these techniques and their impact on FA research) to visualise the nanoarchitecture of both focal adhesions and the forces they exert on the substrate. In this context, it will be especially interesting to include live cell data of actively migrating cells to compare the fork shapes in FAs in the leading to those in the trailing edge.

## MATERIALS AND METHODS

### Cell culture

U2OS cells were kindly provided by Dr. Maarten W. Paul (Erasmus MC, the Netherlands) and previously used for instance in Sánchez et al., 2017, Legerstee et al., 2019 and Legerstee et al., 2021. Cells stably expressing GFP-tagged paxillin (vector previously described in Legerstee et al., 2019) were cultured in Phenol-Red-free DMEM (Lonza) at 37°C and 5% CO2. Culture media were supplemented with 10% FCS (Gibco), 2 mM L-glutamine, 100 U/ml penicillin, 100 µg/ml streptomycine and 100 µg/ml G418. The cell lines are frequently tested for contamination as part of good laboratory practice routine in our lab.

### Sample preparation

For experiments Indium-Tin-Oxide (ITO) coated glass coverslips (22×22×0.17 mm, Optics Balzers) were coated overnight at 4°C with PureCol bovine collagen type I (Advanced Biomatrix) at a final concentration of 10 µg/ml. Cells were seeded onto coated coverslips and maintained for another 36 h at 37°C and 5% CO2. Cells are chemically fixed with 3.75% paraformaldehyde for 12 min and permeabilised with 0.2% Triton-X100 for 10 min, before and after each step the cells were washed thrice with PBS. We note that the formaldehyde fixatives penetrate the cells rapidly but may be extracted by repeated washing. We used this procedure in order to preserve the fluorescence in the cells. The more commonly used osmium tetroxide fixate quenches the fluorescence, while glutaraldehyde, which would cross-link more permanently than paraformaldehyde, is auto-fluorescent. As documented in the manuscript, we found evidence for the appearance of the fork-like FA structure in EM images in literature under conditions where stronger fixation was used. Cells were stained with CF405M Phalloidin (Biotium) following the manufacturer's protocol. Just prior to imaging, the samples were dehydrated using an ethanol sequence (2 min in 70%, 90%, 95%, and 100% ethanol successively).

### Imaging

Integrated fluorescence microscopy and SEM were carried out using a SECOM integrated microscope (Delmic) retrofitted to a SEM (FEI Verios). The SECOM was equipped with an LED light source (Lumencor Spectra), which was used at 405 nm and 475 nm for Phalloidin and GFP respectively with 140 mW total excitation power. Emission was detected using a multi-band filter (Semrock LED-DA/FI/TR/Cy5-4X-A-000) and a CCD camera (Andor Zyla). Exposure time was typically set at 1 s. The ITO cover slide was mounted to the bottom of a SECOM sample holder ring using carbon tape. An extensive overview of imaging procedure in the integrated microscope was published previously (Peddie et al., 2014). Fluorescence images were recorded after closing the SEM chamber but before vacuum pump down. After recording the fluorescence images, the SEM was pumped to high vacuum mode and SEM images of selected regions of interest based on fluorescence expression were acquired. The sample was positioned at a working distance of 4.3 mm and back-scattered electrons were detected using a concentric backscatter detector (CBS). Images (4096×3775 pixels) were recorded at 2 keV electron energy, a current of 1.6 nA, and 10 µs pixel dwell time. Images in Fig. 1 and the top and bottom images on the left side in Fig. 2A were recorded at 3 keV energy and 0.4 nA.

### Analysis

To make overlay images, the EM image was opened in black and white as the background layer in Adobe Photoshop. The fluorescence image is also opened in Photoshop, scaled to the same size as the SEM image (based on the known pixel dimensions) and placed as a separate layer in colour on top of the SEM background layer. The precise overlay is done by hand, mainly based on the F-actin network which due to phalloidin staining is clearly visible in both imaging dualities. To determine the prevalence of fork-shaped, high-contrast areas (defined as any high-contrast area that is partially intersected by an area of lower contrast, splitting the high-contrast area into two sides) in the FAs of our data set all FAs were assigned a number. In total 122 FAs in ten cells (ranging from seven to 15 FAs per cell) were imaged during three independent experiments and were analysed based on the fluorescent images. Of these, 97 FAs (from all ten imaged cells) were also clear enough in the SEM image to be able to determine the shape of the high-contrast areas. The reasons to classify an FA as not clear enough in the SEM image to be able to determine the shape of the high contrast areas were, in order of occurrence: (1) the FA lies too far away from the edge of the cell where the cell is thin enough to image through with the SEM so the FA is lost in the signal coming from the cytoplasm/cytoskeleton of the cell, (2) the FA is too small in the SEM image so while it is possible to make out the FA in the SEM image the resolution of the SEM image is not sufficient to allow the shape of the high contrast area to be determined, or (3) the FA itself or the connecting F-actin fibre is not visible in the SEM image which doesn't allow anything to be said about the shape of a putative darker area at the place where the actin connects to the FA as this connection point is not visible. Of these 97 FAs, two researchers independently counted the number of high-contrast areas and the number of high-contrast areas with a Y-shape, with very similar results: 95 FAs had a high-contrast area and for 71/72 FAs this area was fork shaped, we reported the average. Of the 97 imaged FAs, 73 FAs were classified by one or both observers as fork-shaped, of which two were uniquely identified by observer one and two uniquely by observer two, the remaining 93 FAs were placed in the same category (with/without fork shape) by both observers. To allow a more quantitative analysis of these fork shapes, all fork shapes and the longest axis of the FAs were also traced by hand using the Fiji inbuilt angle and line-drawing and measuring tools. Care was taken that for the angle measurements using the angle measurement tool only the beginning of the fork shape was used.

### Expansion microscopy

Expansion of cultured U2Os cell was performed according to the protocol from M'Saad and Bewersdorf (2020). In short, unstained U2Os cells were fixed with 4% paraformaldehyde for 15 min., washed with PBS and post-fixed with a 0.7% paraformaldehyde/1% acrylamide solution. The fixed cells were embedded in a thin layer of gel (19% Sodium Acrylate, Chem Cruz, catalogue number sc236893; 10% Acrylamide, Sigma-Aldrich, catalogue number 1.19784.0100; 0.1% N,N′-Cystamine-bisacrylamide, Sigma-Aldrich catalogue number 294381; 0.25% Ammonium persulfate, Fisher BioReagents, catalogue number 87687; and 0.25% N,N,N,N′-tetramethylethylenediamine, Sigma-Aldrich, catalogue number A4929).

The gel was allowed to polymerize for 1 h at 37°C. The cells in the gel were denatured in 1 ml denaturation buffer (200 mM SDS, 200 mM Sodium Chloride, 50 mM Tris-HCl pH 6.8) at 73°C for 1 h. After denaturation, all proteins in the gel were stained with 20 μg/ml NHS-ATTO594 (Sigma-Aldrich, catalogue number 08741) in 100 mM bicarbonate for 1.5 h. The gel was expanded in milli-Q water overnight. Afterwards the Gel was expanded in milli-Q water overnight. The resulting cells were expanded approximately seven times.

## Supplementary Material

10.1242/biolopen.059417_sup1 Supplementary information

## Data Availability

Data from this study will be provided by the authors on request.
