# Supplementary material for: Correlative light and electron microscopy reveals fork-shaped structures at actin entry sites of focal adhesions
Source: Biol Open. 2022 Nov 21;11(11):bio059417. doi: 10.1242/bio.059417 (PMC9836080; doi:10.1242/bio.059417)
Supplement: Supplementary information [file biolopen-11-059417-s1.pdf]

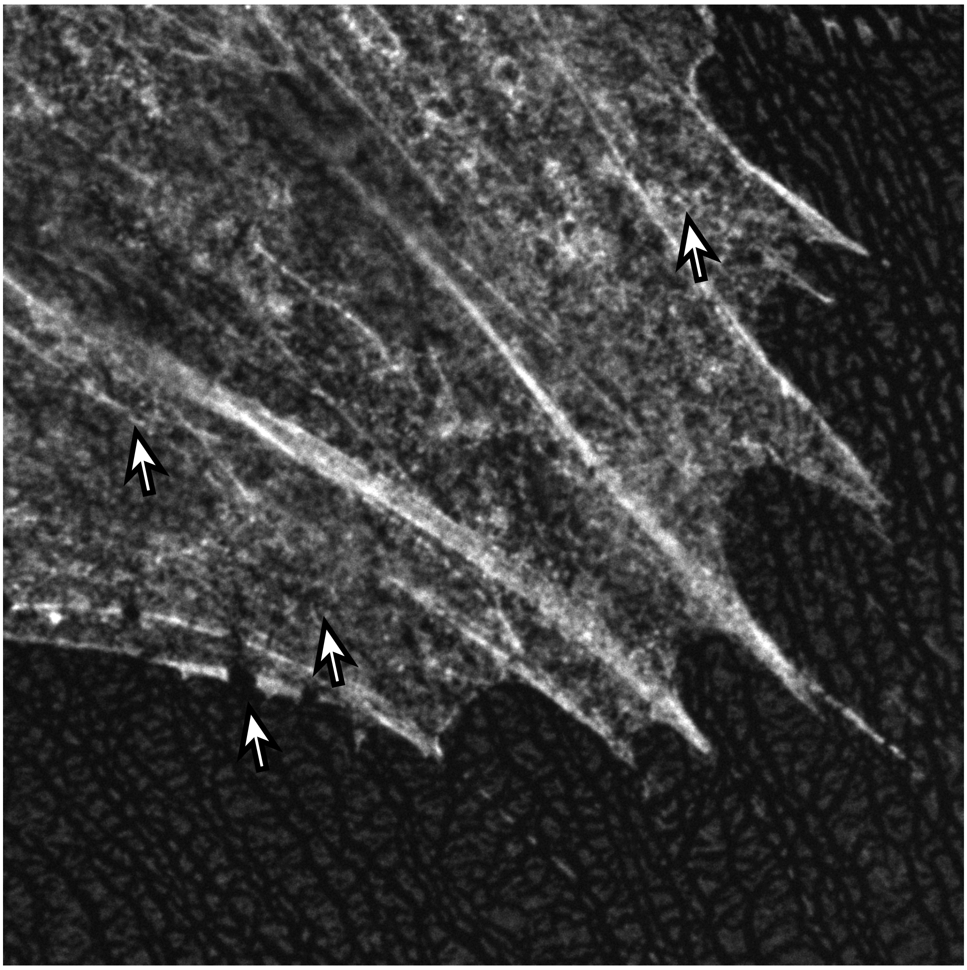

Fig. S1. Confocal image of an expanded U2OS cell (estimated expansion: ~seven times) stained for protein content by PAN staining (see Materials en Methods). The cell was imaged using a Leica SP8 microscope equipped with an 86x, 1.2NA HC PL APO water objective with a motorized coverslip correction ring, which was used to correct aberrations by the gel and the coverslip. To visualise the ATTO-dye, excitation laser wavelength was 594 nm and emission was detected using a 605- 655 nm band pass filter. The arrows indicate fork structures very similar to those observed by EM.
